# Prelamin A accumulation and stress conditions induce impaired Oct-1 activity and autophagy in prematurely aged human mesenchymal stem cell

**DOI:** 10.18632/aging.100651

**Published:** 2014-04-06

**Authors:** Arantza Infante, Andrea Gago, Garbiñe Ruiz de Eguino, Teresa Calvo-Fernández, Vanessa Gómez-Vallejo, Jordi Llop, Karin Schlangen, Ane Fullaondo, Ana M. Aransay, Abraham Martín, Clara I. Rodríguez

**Affiliations:** ^1^Stem Cells and Cell Therapy Laboratory, BioCruces Health Research Institute, Cruces University Hospital, Barakaldo 48903, Spain; ^2^Genome Analysis Platform, CIC bioGUNE & CIBERehd, 48160 Derio, Spain; ^3^Molecular Imaging Unit, CIC biomaGUNE, Paseo Miramón 182 C, 20009, Donostia-San Sebastian, Spain; ^4^Radiochemistry Department, Molecular Imaging Unit, CICbiomaGUNE, Paseo Miramón 182 C, 20009, Donostia-San Sebastian, Spain

**Keywords:** human mesenchymal stem cells, aging, prelamin A, human aging models, Oct-1, autophagy

## Abstract

Aging, a time-dependent functional decline of biological processes, is the primary risk factor in developing diseases such as cancer, cardiovascular or degenerative diseases. There is a real need to understand the human aging process in order to increase the length of disease-free life, also known as “health span”. Accumulation of progerin and prelamin A are the hallmark of a group of premature aging diseases but have also been found during normal cellular aging strongly suggesting similar mechanisms between healthy aging and *LMNA*-linked progeroid syndromes. How this toxic accumulation contributes to aging (physiological or pathological) remains unclear. Since affected tissues in age-associated disorders and in pathological aging are mainly of mesenchymal origin we propose a model of human aging based on mesenchymal stem cells (hMSCs) which accumulate prelamin A. We demonstrate that prelamin A-accumulating hMSCs have a premature aging phenotype which affects their functional competence *in vivo*. The combination of prelamin A accumulation and stress conditions enhance the aging phenotype by dysregulating the activity of the octamer binding protein Oct-1This experimental model has been fundamental to identify a new role for Oct-1 in hMSCs aging.

## INTRODUCTION

Aging, the major risk factor in developing many chronic conditions is characterized by deterioration in the maintenance of homeostatic processes over time, leading to functional decline, with a consequent increased risk for disease and ultimately death. Alteration of the nuclear lamina, one of the cellular changes observed in physiological aging [1] [2], is the hallmark of a group of premature aging diseases known as progeroid laminopathies. These diseases, such as Hutchinson-Gilford Progeria syndrome (HGPS) or *LMNA*-linked lipodystrophy are caused by mutations in *LMNA* gene which encode several isoforms of A-type lamins, being among them Lamin A protein [3, 4], or in *FACE1/Zmpste24* gene which encode a protease essential for the normal processing of Lamin A protein [5]. The consequence of these mutations is the defective maturation of Lamin A, which results in the accumulation of progerin or prelamin A, mutant and immature forms of Lamin A protein respectively. These pathological accumulations at the nuclear envelope cause severe alterations in nuclear morphology and organization, hampering the normal functions of cells and leading ultimately to premature aging phenotypes exhibited by affected patients [6].

Several studies have demonstrated that there is also accumulation of progerin [1] or prelamin A [2] in normally aging cells. Moreover, in a recent study Miller and collaborators have revealed that the presence of progerin is sufficient to induce an aged status in induced Pluripotent Stem Cells (iPSCs) derived differentiated cells, resulting in an interesting strategy for modelling late-onset disease [7]. Nevertheless to date, the molecular mechanisms controlling physiological or pathological aging in the context of progerin and/or prelamin A accumulation and therefore the development of the associated diseases are not fully understood. In the case of HGPS or *LMNA*-linked lipodystrophy, the affected tissues are mainly of mesenchymal origin [8] suggesting that the cell-type specific pathologies in aging could be due, in part, to mesenchymal stem cell (hMSCs) exhaustion or mesenchymal lineage differentiation defects [9, 10, 11].

Another characteristic of aging organisms is that there is decreased autophagy, a basic mechanism of degrading unnecessary or dysfunctional cell components. In fact, the age-related deterioration of autophagic activity results in the accumulation of cellular debris detrimental to cells [12]. Despite its importance in cell physiology, the role of autophagy in mesenchymal stem cells is unknown [13], much less whether the accumulation of progerin or prelamin A could alter the autophagy process in these cells.

Oct-1, a member of the POU domain transcription factor family [14, 15] which is ubiquitously expressed [16] is implicated in diverse biological processes such as housekeeping gene expression [17], early embryonic development [18], and the immune/inflammatory response [19]. Interestingly, some studies have described Oct-1 association with the nuclear lamina through an interaction with Lamin B1, regulating the expression of genes associated with aging and oxidative stress [20, 21]. Although recent evidence describes essential roles for Oct-1 in promoting stemness [22], the role of Oct-1 in mesenchymal stem cell biology has not been determined.

Our group has generated previously a *LMNA*-linked experimental model based on human mesenchymal stem cells treated with a human immunodeficiency virus (HIV) protease inhibitor to induce prelamin A accumulation, which recapitulates the phenotypes observed in biopsies of *LMNA*-linked lipodystrophic patients [23]. Herein we have characterized and evaluated this experimental model as an *in vitro* system for modelling human aging. These prelamin A-accumulating hMSCs (prelamin A-hMSCs) clearly display a premature aging phenotype which affects their functional competence *in vivo*. Moreover, we have found that the combination of prelamin A accumulation and stress conditions enhance the aging phenotype in hMSCs by dys-regulating Oct-1 transcription factor activity. This human aging experimental model has been fundamental for the identification of a new role for Oct-1 in the inhibition of senescence and autophagy and in regulating mTOR signalling pathway in human mesenchymal stem cells, demonstrating their value as a model of human cellular aging.

## RESULTS

### Telomere shortening and increased DNA-damage signalling in prelamin A- accumulating hMSCs

**Figure 1:**
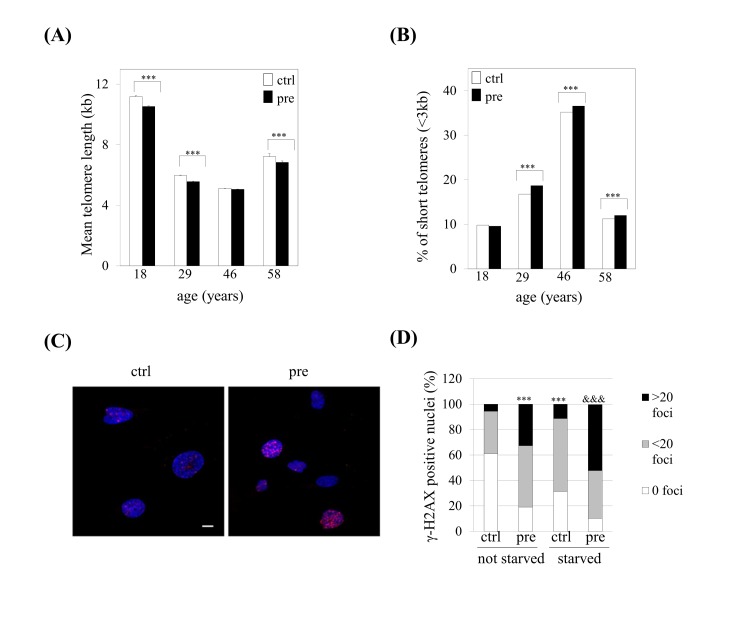
Prelamin A accumulation induces shorter telomeres and increased DNA damage signalling in hMSCs. (**A**) Telomere length and (**B**) the percentage of short telomeres were analyzed by HT-Q-FISH. At least 17,000 nuclei were analyzed per sample. Bars are average +/- standard deviation of 3 independent donors. (**C**) Representative immunofluorescence micrograph and (**D**) quantification of γ-H2AX foci. Blue: DAPI, red: γ-H2AX. Scale bar: 10 μm. At least 100 nuclei were analyzed per sample. χ^2^ was used for statistical significance. *** p<0.001 when compared *versus* ctrl-hMSCs. &&& p< 0.001 when compared *versus* ctrl-hMSCs starved. (pre): prelamin A-accumulating hMSCs, (ctrl): control-hMSCs.

Our previous studies provided evidence that prelamin A accumulation in hMSCs leads to reduced lifespan in culture as a consequence of premature senescence of these cells [23]. To assess whether we could take advantage of this system as an experimental model of human cellular aging, we challenged prelamin A-hMSCs to recapitulate other hallmarks of aging recently proposed by Lopez-Otín and collaborators [24]. We first studied whether prelamin A accumulation can induce telomere shortening, a characteristic of normal aging cells. We analyzed telomere length in control (ctrl) or prelamin A-hMSCs (pre) from four different bone marrow donors of different ages (Fig. 1A) using a quantitative fluorescence *in situ* hybridization, HT-Q-FISH [25]. As shown in Figure 1A, hMSCs had an average telomere length ranging from 5.11 to 11.17 kb, in agreement with previous studies in which a mean telomere length of 7.2 kb has been described for adult hMSCs [26]. As expected, the youngest donor (18 years of age) had the longest telomeres (11.17 kb in control cells). Of note, we observed in each donor a decline in mean telomere length of prelamin A-hMSCs when compared to the controls cells, a change which was statistically significant in three samples (640 bp loss in 18 year old donor, 400 bp loss in 25 year old donor, 380 bp loss in 58 year old donor). Given that the percentage of critically short telomeres in human cell population increases significantly with age [27, 25] we explored whether prelamin A accumulation induced such increase in hMSCs *in vitro*. The percentage of telomeres with a length < 3kb was slightly but significantly increased (except for the 18 years old donor) in prelamin A-hMSCs (Fig. 1B).

Given the connection that the shortening of telomeres has with DNA damage [24], we wondered whether prelamin A accumulation in hMSCs could induce the activation of the DNA damage response. The phosphorylation status of the histone H2AX (γ-H2AX), a long standing marker of DNA damage, was analyzed obtaining that prelamin A accumulation in hMSCs induced an increased activation of DNA damage signalling comparing with their control counterparts (Fig 1C and 1D left columns).

Aged cells are hypersensitive to stress conditions due to defects in their stress response pathways. Thus, we wondered whether stress conditions, such as serum starvation, could enhance this increased DNA damage response as a consequence of prelamin A accumulation in hMSCs. As shown in Figure 1D, control-hMSCs which had been submitted to serum starvation, showed a significant increase in the percentage of nuclei which presented γ-H2AX foci when compared to control cells. This percentage was significantly higher in prelamin A-hMSCs (80%) when compared to control cells (40%) (Fig. 1D, right columns). Moreover, the combination of prelamin A accumulation and serum starvation conditions led to a greater increase in γ-H2AX signalling, in which almost 50% of cells that had more than 20 foci were observed (Fig. 1D).

### Prelamin A accumulation and stress conditions induce a decrease in cell survival and impaired autophagy in human mesenchymal stem cells

**Figure 2:**
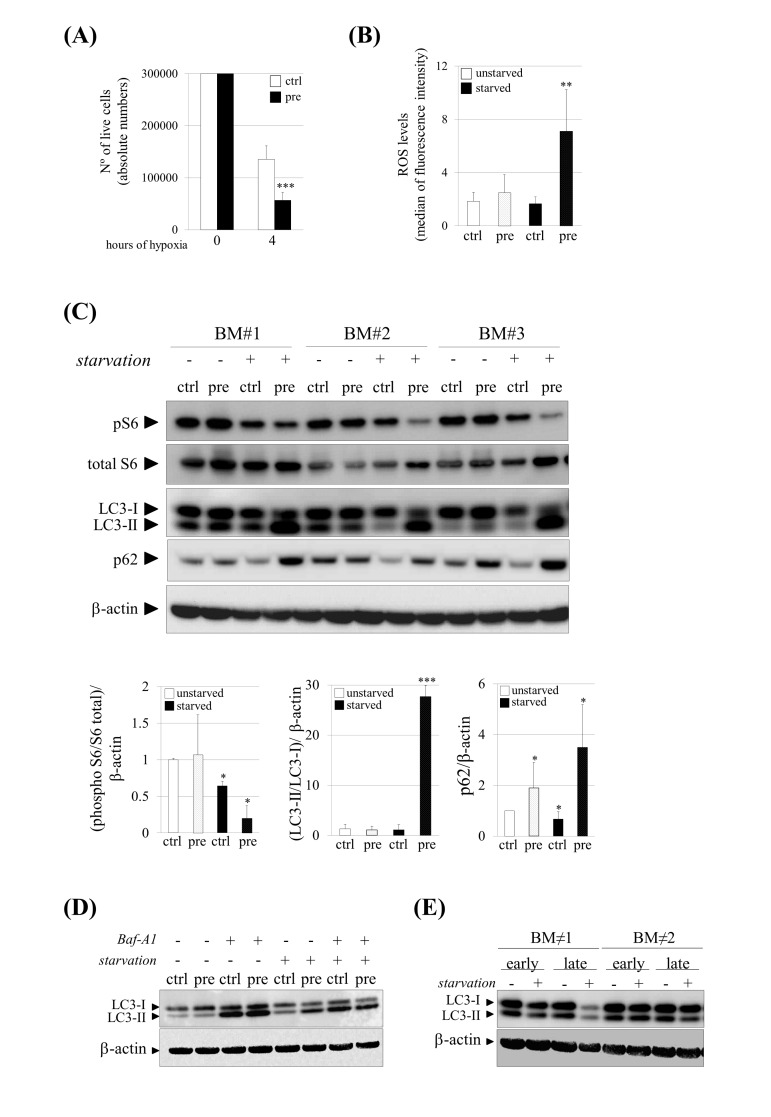
Increased susceptibility and impaired autophagy under stress conditions in pre-hMSCs. (**A**) Number of live cells after submitting hMSCs to hypoxia. (**B**) Reactive Oxygen Species (ROS) measurement of hMSCs cultured under basal (unstarved) or starvation conditions (starved). (**C**) Western blot of indicated proteins in ctrl and pre-hMSCs cultured under basal or serum starvation conditions. β-actin was used as loading control. hMSCs from three independent bone marrow donors (BM) where analyzed together. Densitometry for each immunoblot is provided. (**D**) Determination of autophagic flux of hMSCs treated with Bafilomycin (Baf-A1) 0.1 μM during 9 hours. LC3-I and LC3-II expression levels are shown and β-actin was used as loading control. (**E**) Western blot of LC3-I and LC3-II expression levels in replicative senescent hMSCs (Early: early-passage hMSCs; Late: late-passage hMSCs), Bars are average +/- standard deviation of 3 independent donors. Differences marked with asterisks are significantly different from control unstarved (panel A and D) or control starved cells (panel B) *** p<0.001, ** p<0.01, *p<0.05. (pre):prelamin A-accumulating hMSCs, (ctrl):control-hMSCs.

To address the question whether prelamin A accumulation in hMSCs could cause an increase susceptibility under stress conditions we analyzed their survival after incubations in sudden hypoxic environments in which hMSCs must face an abrupt stress situation. Cell survival assays in the presence of hypoxia during 4 hours showed that approximately 50% of control cells survived while only 20% of hMSCs which accumulated prelamin A did it (Fig. 2A), confirming that prelamin A-hMSCs are less resistant to stress conditions.

As oxidative stress activated by the sustained production of oxidant molecules is also associated with aging and related diseases [28], we wondered whether prelamin A accumulation could increase Reactive Oxygen Species (ROS) levels in hMSCs. As shown in Figure 2B, although there were no differences in ROS levels between control and prelamin A-hMSCs, a significant increase of ROS was detected in prelamin A-hMSCs when cultured under starvation conditions.

Autophagy, a process by which cellular proteins and damaged organelles are degraded, is reduced or impaired in aging cells [29, 30]. We first evaluated the activation of mTORC1, a major negative regulator of autophagy [31] through the phosphorylation status of one of its downstream target substrates [32], the ribosomal protein subunit S6 (S6). Consistent with serum deprivation conditions, known to be a stress signal that activates autophagy [33], phospho-S6 protein (pS6) was down-regulated in control-hMSCs when compared to cells which had been cultured under normal conditions, reflecting the inhibition of mTORC1, as expected (Fig. 2C). Surprisingly, under prelamin A accumulation and serum starvation conditions, the mTORC1 pathway was even more repressed than in serum starved control cells (Fig. 2C), suggesting that autophagy could be enhanced in prelamin A-hMSCs under those stress conditions. To test this possibility we analyzed the lipidation status of the LC3 protein. During autophagy the C-terminal extension of the cytosolic protein LC3, is cleaved to form the processed LC3-I, which is finally modified into the phosphatidylethanolamine-conjugated form, known as LC3-II being then recruited to auto-phagosome membranes [34]. In prelamin A-hMSCs cultured under serum starvation conditions (Fig. 2C), there was a remarkable shift from LC3-I to the LC3-II indicating an increase in autophagosome formation. Because an increase in LC3-II abundance could be due to either an increase of autophagic flux or blocked autophagosome maturation, we quantified the amounts of p62, a protein that binds polyubiquitinated proteins that is degraded during the autophagic process, and therefore used as a marker of autophagic flux. As expected, p62 protein expression decreased in control-hMSCs under serum starvation conditions. However, in prelamin A-hMSCs under serum starvation conditions, a striking increase in p62 protein levels could be detected, indicating that autophagic flux was inhibited or delayed in these cells This observation was further confirmed by treating hMSCs with Bafilomycin A1 (Baf-A1), a lysosomal proton pump inhibitor which causes the accumulation of autophagosomes and therefore an increase in LC3-II levels when compared to untreated cells. As expected, we did not detect any difference between unstarved ctrl and pre-hMSCs after Baf-A1 treatment, but under serum starvation conditions Baf-A1 treated pre-hMSCs failed to accumulate LC3-II, contrarily to what occurred in control cells (Fig. 2D) Of note, there was a slight but significant increase in p62 expression in prelamin A-hMSCs cultured under normal conditions (Fig. 2C), suggesting that prelamin A itself induces the expression of this protein.

Finally, we confirmed the reduction of autophagy from a general perspective of aging such as hMSCs in replicative senescence. For that, we compared early-passage hMSCs (<5) with late-passage ones (>15) under both basal and serum starvation conditions detecting a decrease in LC3-II levels at late passages cells only after serum starvation conditions (Fig. 2E).

Overall, these results confirm that autophagy is reduced or impaired in senescent hMSCs.

### hMSCs which accumulate prelamin A show a diminished functional activity *in vivo*

**Figure 3:**
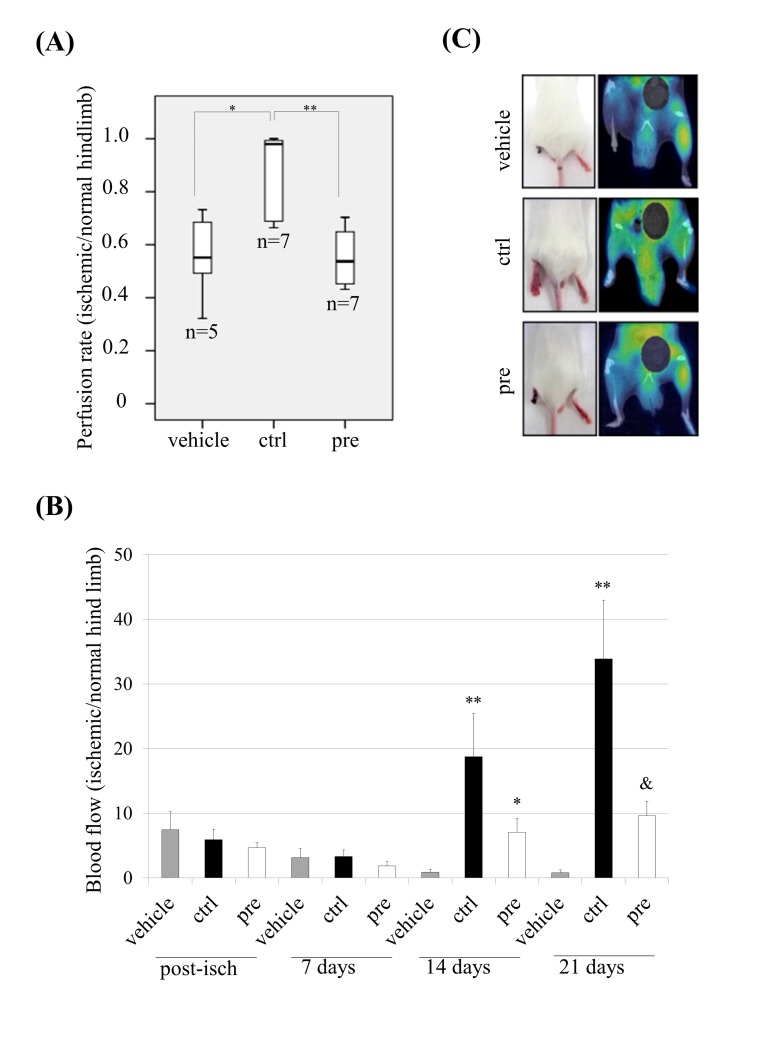
Dys-functional activity of hMSCs which accumulate prelamin A *in vivo*. (**A**) Perfusion rate in mice after transplanting hMSCs. ** p<0.01, * p<0.05. (**B**) Laser-Doppler quantification of hind limb blood flow over time after hMSCs transplantation (Post-isch = blood flow measured immediately after ischemia). ** p<0.01 and * p<0.05 when compared *versus* vehicle, & p<0.05 when compared *versus* control hMSCs. (**C**) Representative photographs of animal hind limbs (left) and PET images (right) 14 days after ischemia and hMSCs transplantation. Bars are average +/- standard deviation (panel A) or standard error of the mean (panel B). (pre): prelamin A-accumulating hMSCs, (ctrl): control-hMSCs.

Since prelamin A-hMSCs recapitulate all the aged phenotypes tested, we decided to ascertain the relevance of these changes by challenging their functional competence *in vivo*. One of the established models for this purpose is based on the ability of hMSCs to significantly improve vascular circulation after their transplantation into the ischemic hind limbs of immunocompromised mice [35]. Thus, mouse limb ischemia was induced by ligation of the femoral artery and its branches in the left hind-limb of SCID mice. Then, control (ctrl) or prelamin A-hMSCs (pre) were transplanted by intramuscular injection into the left hind-limb immediately after ligation. As a control of the technique, a subset of mice was injected with culture medium alone (vehicle). 14 days after cell transplantation, the perfusion rate (tissue functionality) of the control and the ischemic limbs was assessed by Positron Emission Tomography (PET). The vehicle group (n=5) presented a mean perfusion rate of 0.5566 (standard deviation: 0.1632) and similarly, the set of mice given an injection of prelamin A-hMSCs (n=7) exhibited a mean of perfusion rate of 0.5534 (standard deviation: 0.113) (Fig. 3A). However, animals treated with control-hMSCs (n=7) significantly improved their perfusion rate (mean 0.8580; standard deviation: 0.1666) in contrast to the vehicle group (p<0.05) or prelamin A-hMSCs group (p<0.01). We also monitored the hind limb blood flow with a laser-Doppler system over time: post-ischemia and 7, 14 and 21 days after transplantation, comparing the blood flow in the ischemic hind limb versus the blood flow in the contralateral one. Laser Doppler revealed a significant restoration of blood flow after 14 days post-ischemia in limbs injected with control-hMSCs (Fig. 3B). Consistent with these results, 100% of mice injected with vehicle lost their left hind limb and 60% of animals injected with prelamin A-hMSCs did it. However, only 28% of mice injected with control-hMSCs suffered the amputation of hind limb as a consequence of the induced ischemia (Fig. 3C).

### hMSCs under prelamin A accumulation and serum starvation conditions exhibit a gene expression signature and phenotype of senescence

The results obtained under stress conditions suggested that serum starvation could induce an altered gene expression profile in prelamin A-hMSCs. To assess this supposition, we performed a whole genome, microarray-based gene expression analysis of prelamin A-hMSCs compared to control cells cultured under serum starvation. Significant changes (fold ±1.4, p<0.05) in more than 1000 transcripts were found being approximately 50% of the changes up-regulation and the other 50% down-regulation.

In an attempt to address the functional relevance of these differentially expressed genes, gene ontology (GO) enrichment analysis was carried out for the up and the down-regulated genes using the DAVID bioinformatic resource [36]. Interestingly, among the most over-represented categories within the up-regulated transcript list were genes related to cell proliferation, lysosomes, aging, response to oxidative stress and mitochondria, whereas among the down-regulated transcripts, the most significant enriched categories were related to the cytoskeleton, cell adhesion and oxidation reduction (Table 1). These results showed that prelamin A-hMSCs under serum starvation conditions exhibit gene expression characteristics of senescent hMSCs which are known to present changes in cytoskeleton, cell adhesion and oxidative stress [37]. Moreover, the changes in the expression of genes related to aging and lysosomes categories strengthened this observation since we have detected hallmarks of aging in these cells and alterations in their autophagy.

**Table 1:** Significantly enriched categories among the up-regulated and down-regulated genes using Gene Ontology analysis by DAVID

	GO ACCESSION	GO Term	P value
*Up-regulated transcripts*	GO:0042127	regulation of cell proliferation	0.0004
	GO:0005764	lysosome	0.013
	GO:0031982	vesicles	0.02
	GO:0007568	aging	0.03
	GO:0005739	mitochondrion	0.034
	GO:0006979	response to oxidative stress	0.035
	GO:0016209	antioxidant activity	0.036
*Down-regulated transcripts*	GO:0005856	cytoskeleton	0.000004
	GO:0007155	cell adhesion	0.0001
	GO:0030198	extracellular matrix organization	0.001
	GO:0055114	oxidation reduction	0.01

**Figure 4:**
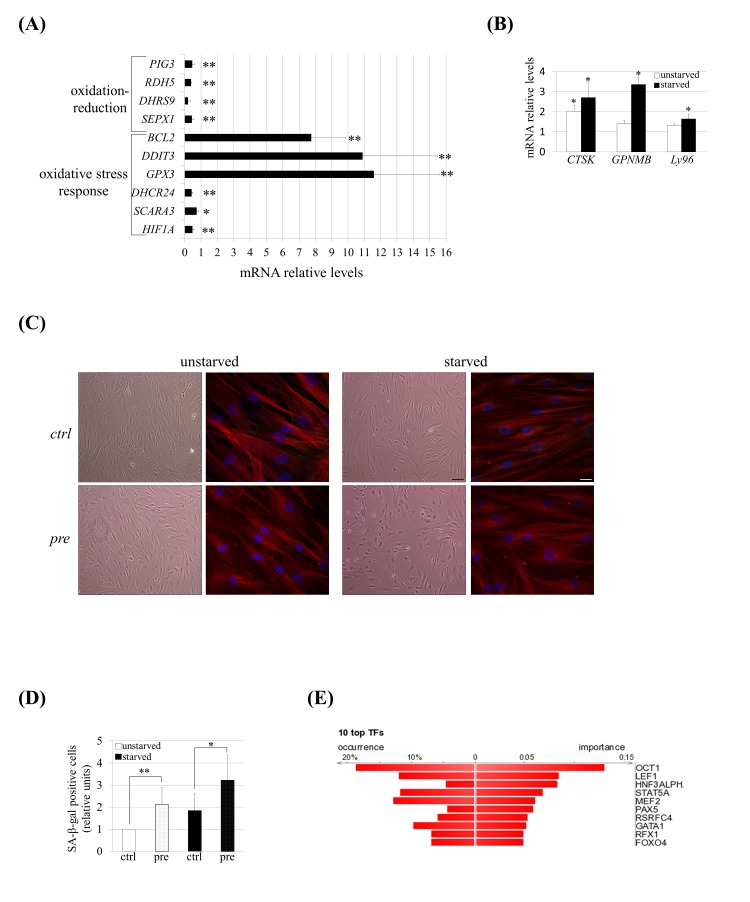
hMSCs show an altered transcriptomic profile and phenotype of senescence under prelamin A accumulation and serum starvation conditions. (**A**) Q-RT-PCR validation for a subset of genes grouped in oxidation-reduction and response to oxidative stress categories and (**B**) for genes known to be up-regulated in senescent hMSCs. The gene expression ratio for pre-hMSCs *versus* control-hMSCs under starved conditions is shown in A and in the case of B it has been included too the comparison of pre-hMSCs *versus* ctrl-hMSCs ratio under unstarved conditions. For gene expression normalization *GAPDH* was used. Bars are mean +/- standard error mean of 3 independent donors. ** p< 0.01, * p<0.05. (**C**) Senescence-associated morphological changes in pre-hMSCs under serum starvation conditions. Bright field images (scale bar: 100 μm) and confocal immunofluorescence images (scale bar: 20 μm) are shown. Red: beta-tubulin, blue: DAPI. (**D**) SA β-gal quantification in hMSCs under basal (unstarved) or serum starvation conditions (starved). Bars are average +/- standard deviation. ** p< 0.01, * p<0.05. (**E**) DiRE analysis of genes found to be dys-regulated in pre-hMSCs. The graph shows the top 10 candidate transcription factors ranked by importance. (pre): prelamin A-accumulating hMSCs, (ctrl): control-hMSCs.

The gene expression dys-regulation was validated (Fig. 4A) by performing Q-RT-PCR analysis on a subset of dys-regulated genes included in oxidation-reduction (*SEPX1*, *DHRS9*, *RDH5* and *PIG3*) and in oxidative stress response (*HIF1A*, *SCARA3*, *DHCR24*, *DDIT3*, *BCL2* and *GPX3*) categories identified previously by DAVID analysis (Table 2). We also validated the expression of some genes known to be up-regulated in senescent hMSCs [38]: *CTSK*, *GPNMB* and *Ly96* (Table 2 and Fig. 4B). At the same time, we detected typical morphological changes confirming the enhanced senescence of these cells: pre-hMSCs under serum starvation became larger with irregular and flat shape (Fig. 4C) and these cells exhibited increased senescence associated β-galactosidase (SA-β-gal) staining (Fig. 4D).

**Table 2:** Name, functional role, dys-regulation and description of the dys-regulated genes validated by Q-RT-PCR analysis

Name	Gene Ontology	Up/Down	Gene Description
SEPX1	Oxidation-Reduction	Down	Methionine-R-sulfoxide reductase B1
DHRS9		Down	Dehydrogenase/Reductase 9
RDH5		Down	Retinol dehydrogenase 5 (11-cis/9-cis)
PIG3		Down	P53-inducible gene 3 quinone oxidoreductase
HIF1A	Oxidative Stress Response	Down	Hypoxia inducible factor 1, alpha subunit
SCARA3		Down	Scavenger receptor class A, member 3 / Cel Stress Resp1
DHCR24		Down	24-dehydrocholesterol reductase
DDIT3		Up	DNA-damage-inducible transcript 3
BCL2		Up	B-cell CLL/lymphoma 2
GPX3		Up	Glutathione peroxidase 3
CTSK	Aging	Up	Cathepsin K
GPNMB		Up	Glycoprotein (transmembrane) nmb
LY96		Up	Lymphocyte antigen 96

To explore the molecular mechanisms that could be responsible for these enhanced senescence, a bio-informatic program, Distant Regulatory Elements of co-regulated genes (DiRE) [39], was used to determine the transcription factor binding sites that are enriched among the co-expressed dys-regulated genes. Comparing the significantly dys-regulated genes (fold ±1.4) *versus* a random set of 5,000 genes, DiRE showed that Oct-1 was the most strongly over-represented transcription factor that could be governing this altered genetic program (Fig. 4B).

### Oct-1 overexpression and impaired activity in prelamin A-hMSCs under serum starvation conditions

**Figure 5:**
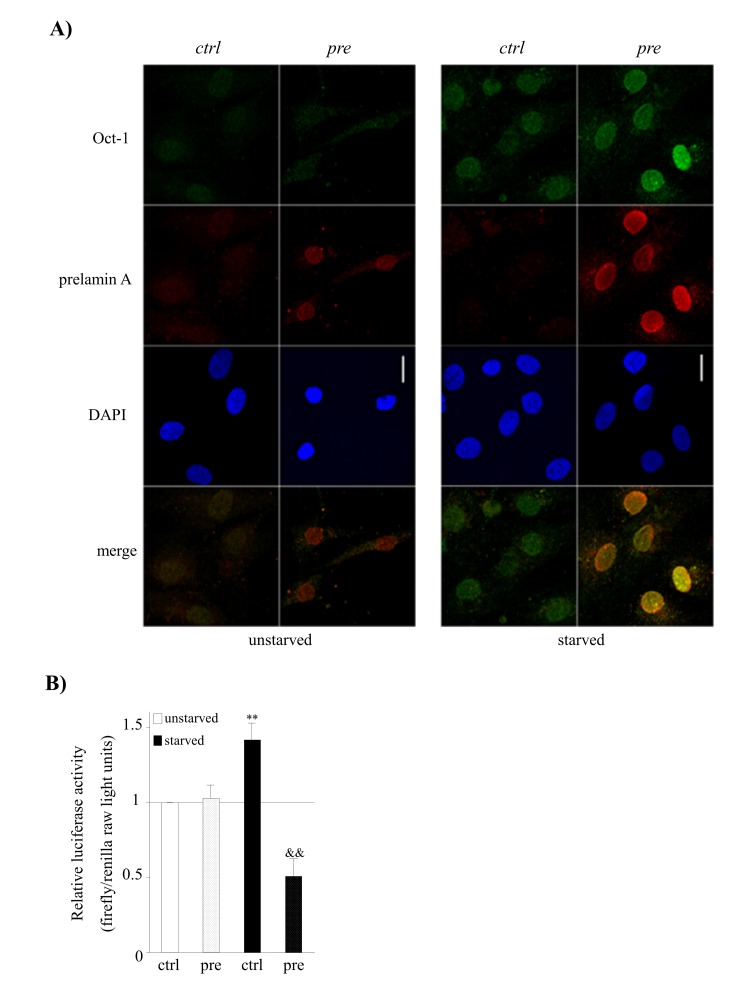
Prelamin A accumulation and serum starvation conditions induce the overexpression of Oct-1 and its impaired activity in hMSCs. (**A**) Representative confocal immunofluorescence staining showing the expression of Oct-1 and prelamin A in hMSCs under basal (unstarved) or serum starvation conditions (starved). Scale bar: 20 μm. (**B**) Luciferase reporter assays of Oct-1 transcription factor activity in hMSCs cultured under under basal (unstarved) or serum starvation conditions (starved). Bars are average +/- standard deviation of 3 independent donors. Differences that are significant are marked as follows: ** p<0.01 when compared to control-hMSCs, && p<0.01 when compared to control-hMSCs starved.

Given that Oct-1 is known to be a sensor of cellular stress [40] we assessed whether serum starvation conditions and/or prelamin A accumulation affects Oct-1 expression and subcellular distribution by confocal microscopy analysis. As expected, we detected that the expression of Oct-1 was induced and its localization was nuclear in control-hMSCs after subjecting the cells to a stress condition such as serum starvation (Fig. 5A).

Although prelamin A accumulation did not induce any alteration in Oct-1 expression, we detected an over-expression of Oct-1 under both serum starvation and prelamin A accumulation circumstances (Fig. 5A). Strikingly, prelamin A accumulation itself was induced in prelamin A-hMSCs under serum starvation conditions (Fig. 5A), revealing that the combination of both, the previous existence of prelamin A and serum starvation conditions, promotes the accumulation of greater amounts of prelamin A by an unknown mechanism.

Subsequently, we tested whether the overexpression of Oct-1 under prelamin A accumulation and serum starvation conditions resulted in an enhancement of its transcriptional activity. As can be seen in Figure 5B, Oct-1 reporter activity significantly increased in control-hMSCs under serum starvation conditions, consistent with its role as a stress sensor and with its increased expression (Fig. 5A). Although prelamin A accumulation itself did not alter the transcriptional activity of Oct-1 (Fig. 5B), surprisingly, this activity was significantly decreased in prelamin A-hMSCs under serum starvation conditions.

### Silencing of Oct-1 results in the induction of senescence and autophagy in human adult stem cells

**Figure 6:**
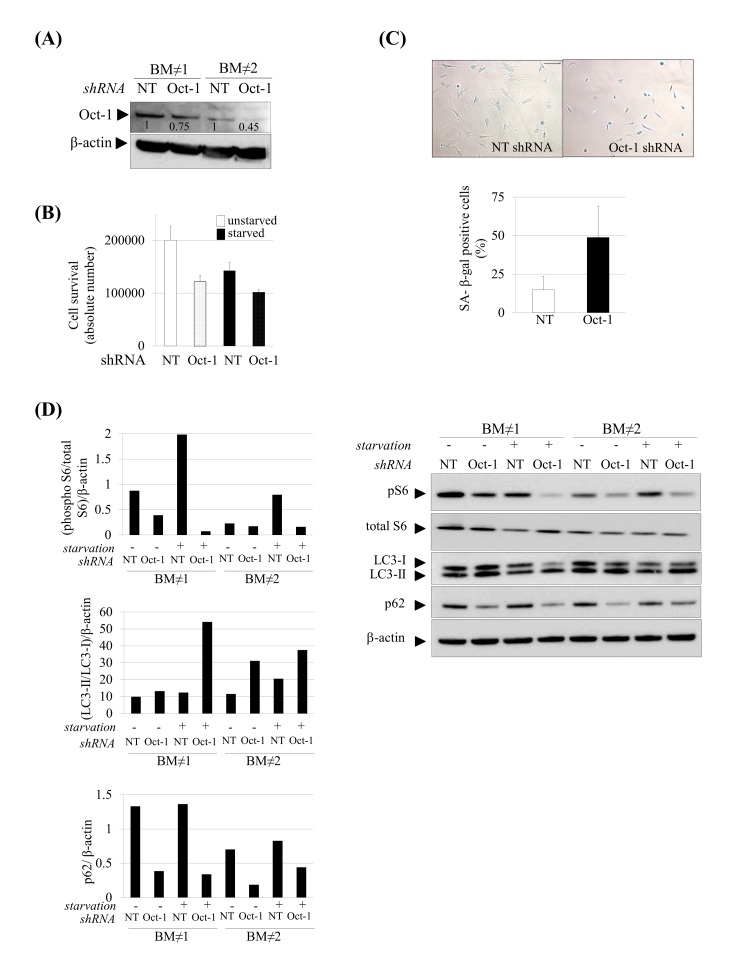
Induction of senescence and autophagy in Oct-1 silenced hMSCs. (**A**) Western blot showing decreased Oct-1 expression after Oct-1 shRNA plasmids transduction in hMSCs, NT: Non Targeting shRNA. Numbers show densitometric quantification for Oct-1 expression. (**B**) Cell survival after Oct-1 silencing in hMSCs cultured under basal (unstarved) or serum starvation conditions (starved). (**C**) Representative SA β-gal staining (left) and quantification (right) in hMSCs transduced with NT or Oct-1 shRNA. Scale bar: 100 μm. (**D**) Western blot of indicated proteins from hMSCs silenced for Oct-1 or not (NT). β-actin was used as loading control. Densitometry for each immunoblot is provided. For these experiments hMSCs from two independent donors were used(BM≠1 and BM≠2). Bars are mean +/- standard deviation

In the previous experiments we found that the dys-regulation of Oct-1 transcriptional activity could account for the altered transcriptomic profile that exhibit prelamin A-hMSCs under serum starvation conditions. In order to elucidate the potential role that Oct-1 plays in hMSCs homeostasis, we decided to suppress its expression in these cells. After confirming the silencing by Western blot analysis (Fig 6A), we analyzed the susceptibility of Oct-1 silenced hMSCs to stress conditions such as serum starvation. As can be seen in Figure 6B, there was an appreciable decrease in cell survival after Oct-1 silencing in hMSCs cultured under normal conditions. Under serum starvation conditions, control-hMSCs infected with non targeting vectors (NT) exhibited a reduction in their cell number, probably due to the fact that the infection process itself makes hMSCs more susceptible to stress conditions. On the contrary, Oct-1 silenced hMSCs under stress conditions exhibited a similar decline of cell survival compared to normal conditions, showing first that the function of Oct-1 in hMSCs is essential for promoting cell survival under normal conditions and second that Oct-1 silencing does not make the cells more susceptible to stress conditions.

Then, we wonder whether Oct-1 could account for other hallmarks of aging focusing on two which were also altered in our experimental human model as a consequence of prelamin A accumulation: cellular senescence and autophagy. Senescent cells were analyzed by β-galactosidase staining (Fig. 6C) which showed a significant increase in senescence in hMSCs silenced for Oct-1. We next assessed the autophagy pathway in Oct-1 silenced cells (Fig 6D). Similar to prelamin A-hMSCs under serum starvation conditions (Fig 2C), there was a decrease in the expression of phospho-S6 protein in Oct-1 silenced cells especially under serum starvation conditions and an increase in the formation of autophagosomes, as denoted by the shift from LC3-I to LC3-II form. These results showed that Oct-1 functions as an essential transcription factor for the activation of the mTOR pathway and inhibition of autophagy under stress conditions. However, and contrary to our previous observations in prelamin A-hMSCs, the autophagy process in Oct-1 silenced hMSCs seemed to be efficient, as denoted by the decreased expression of p62 protein. Importantly, this result was seen independently of serum starvation conditions (Fig 6D), indicating that Oct-1 is a critical factor for regulating p62 expression in hMSCs.

## DISCUSSION

An undeniable fact of our society is that population aging is increasing due to the higher expectancy of life. Given the close relationship between aging and developing diseases such as cancer, neurodegenerative or cardiovascular disorders, there is a real need to understand the molecular mechanisms underlying this inevitable process. The final objective would be to delay aging-associated diseases in order to increase the length of disease-free life, also known as “health span”. The aging process has been studied in various organisms such as nematodes, flies or mice, rendering valuable information, however none of them accurately recapitulate this process in humans [41]. In recent years human model systems have emerged to study cellular aging to overcome this drawback, for example premature aging syndromes associated with the *LMNA* gene [42-44]. The use of these models for studying physiological human aging is justified by the fact that accumulation of progerin in hMSCs of aged individuals [45] and in normal fibroblasts during late-passages of culture [46] has been detected, as well as prelamin A in normal aging vascular smooth muscle cells (VSMC) [2].

It has been previously described in the literature that the accumulation of prelamin A induced by some HIV protease inhibitors leads to a premature senescent phenotype in several cell types, including fibroblasts [47], endothelial or peripheral blood mononuclear cells [48]. In fact, recently we provided evidence that prelamin A accumulation induced by an HIV protease inhibitor, causes a reduced lifespan in culture as a consequence of premature senescence in mesenchymal stem cells [23], an observation which has been confirmed afterwards [49]. In the present work we further explore the senescent phenotype of pre-hMSCs previously detected by our group, and we propose an experimental model of early onset of human aging based on the induced accumulation of prelamin A in adult human stem cells (such as hMSCs). This model shows a less severe phenotype (similar to that observed in *LMNA*-linked lipodystrophy) than the progeria human experimental models and patients present (late onset aging). First, we found that prelamin A-hMSCs presented some characteristics of normal aging cells, such as the shortening of telomeres and the induction of DNA damage. We show for the first time that prelamin A accumulation in hMSCs causes a decrease in telomere length by an unknown mechanism and subsequently an increase in the percentage of short telomeres. Moreover, this decrease seems to be dependent on telomere length per se, since individuals with longer telomeres (18 years old) despite showing greater attrition of telomeres comparing to older individuals, do not show a difference in the relative number of shorter telomeres. Consistent with this observation, individuals older than 18 years of age, showed not only the shortest telomeres, but also had the greatest increase in the percentage of critically short telomeres (< 3 kb) under prelamin A accumulation, a characteristic of aged cells [25]. These results highlight the effects that the accumulation of prelamin A exerts on telomeres length, and specifically to shorten telomeres when they reach a minimum threshold of length in hMSCs. Of note, although the relationship between prelamin A accumulation and telomeres has not been studied in depth, there is controversy in the literature about whether progerin induces or not the shortening of telomeres. This disparity of results between groups probably is due to the fact that different technologies have been used to assess telomere length. However, recent results obtained using the extremely sensitive and accurate HT-Q-FISH (the same technology that we used in our model) point out that progerin induces the shortening of telomeres and an increment in the percentage of short telomeres [7], similarly to what prelamin A does in hMSCs.

Because decreased autophagy is inherent to aging organisms, we hypothesized that prelamin A accumulation could hamper this process in hMSCs. In fact, the impairment of autophagy only under prelamin A accumulation and serum starvation conditions was unexpected, suggesting that this autophagy is inefficient at first, and that it contributes to the premature aging phenotype seen in hMSCs. Supporting this assumption, the observation of impaired autophagy in our model is similar to those obtained in some *LMNA*-related murine models: such as *Lmna* knockout mice (dilated cardiomyopathy and muscle dystrophy) and *Zmpste24*-deficient mice (extreme phenotype of premature aging) [50, 51]. Both murine models share with our human experimental model an increase in LC3-II protein, which suggest that autophagosome formation is enhanced, however there are significant disparities regarding p62 and mTOR pathway activation. These results clearly indicate that alterations in Lamin A homeostasis, such as those observed in progeroid laminopathies, induce the impairment of the autophagy process, although the reasons for these differences among the *LMNA*-related murine phenotypes among themselves and with the prelamin A-hMSC experimental human model are unknown. These discrepancies could be originated by distinct Lamin A roles in different species (highlighting the need of human experimental models to study human diseases) as well as between different cell types (heart, muscle and liver in the case of knockout mice and hMSCs in ours) even in the levels of prelamin A accumulation.

Regarding our model, an unexpected finding was that mTOR pathway is inactivated under prelamin A accumulation and serum starvation conditions. With this result, an induction of autophagy process in these cells is expected, similarly to what occurs when cells are treated with rapamycin, an inhibitor of mTOR. In fact, the link among mTOR pathway inhibition by rapamycin, the induction of autophagy and the reversion of aged phenotype, in HGPS cells [52] as well as in healthy ones [53] has been recently described. This reversion of aged phenotype is thought to be due to the reduced levels of progerin [52, 54] and farnesylated prelamin A [55] induced by rapamycin. However, autophagy process in pre-hMSCs under serum starvation conditions is impaired in spite of the inhibition of mTOR pathway. Further studies with rapamicyn and/or other compounds interfering with mTOR pathway will clarify this unexpected result.

Strikingly, some phenotypes were enhanced (the phosphorylation of H2AX) or revealed (increase in ROS levels, impaired autophagy, deep gene expression changes, Oct-1 overexpression and functional repression) in prelamin A-hMSCs under stress. These facts implied that both the accumulation of prelamin A and serum starvation act synergistically to activate or repress signaling pathways. Remarkably, prelamin A is over-expressed under serum starvation conditions in hMSCs which already accumulate prelamin A. Although the molecular mechanism governing this effect is unknown, we suggest that this prelamin A “over-accumulation” could be the final consequence of the phenotypes that we do not observe under normal conditions. Thus, prelamin A levels would be directly linked with the severity of the premature aging phenotypes that cells exhibit. This hypothesis is supported by a recently published work which demonstrates that the severity of neonatal lethal restrictive dermopathy in which there is prelamin A accumulation, is due to the higher expression of prelamin A compared to the accumulation of progerin in HGPS [56] or even much more than the accumulation of prelamin A in *LMNA*-linked lipodystrophy, which is similar to our model [23].

The relevance of the aging phenotypes exhibited by hMSCs as a consequence of prelamin A accumulation is demonstrated by the reduced functionality of these cells *in vivo*. After injecting them into ischemic limbs of mice, prelamin A-hMSCs could die before they can exert any beneficial effect on the surrounding tissues, similar to what occurs in hMSCs derived from progeria-iPSCs [42]. These results clearly highlight the toxic effect that prelamin A induces in the functionality of hMSCs *in vivo*. Since one characteristic of aging is a decline in the regenerative potential of mesenchymal tissues, these results supports the hypothesis that the homeostasis of hMSCs from individuals suffering premature aging diseases is hampered. It is important to consider that affected tissues in age-associated disorders are not only from mesenchymal origin, but also originate from other tissues such as neuroectoderm (neurodegenative diseases) in contrast to what it is observed in progeroid syndromes. Interestingly, there is parallelism between the mesenchymal tissues affected in age-associated disorders and premature aging diseases arising from the accumulation of progerin or prelamin A. In fact, lipoatrophy, osteopenia, cardiomyopathy and atherosclerosis are frequently detected in aged individuals, as well as in patients with premature aging syndromes associated with *LMNA* gene, such as progeria or *LMNA*-linked lipodystrophy [57]. Taking this into consideration, we propose that prelamin A accumulation in hMSCs contributes to exhaustion of the stem cell pools, which is indeed one of the hallmarks of aging [24] and one of the causes underlying the pathology of aging (physiological or pathological). Must be pointed out that we haven't detected any progerin expression in prelamin A-hMSCs (data not shown) which excludes the possibility that the premature aging phenotype observed in our model could be due to progerin accumulation.

The value of our human experimental aging model has been clearly evidenced by the identification of a novel and fundamental role that Oct-1 plays on hMSCs homeostasis, unknown until now. The experimental model allowed us to delve into the mechanism of aging in hMSCs, leading to the identification of the reduction of Oct-1 transcriptional activity in the process of hMSCs aging associated with prelamin A accumulation. Moreover silencing experiments confirmed the involvement of Oct-1 in regulating the molecular mechanism underlying the premature aging phenotype of hMSCs, such as the dys-regulation of autophagy under serum starvation conditions.

Despite the fact that Oct-1 silenced hMSCs showed an increased autophagy, this phenotype was not identical to that observed in prelamin A-hMSCs under serum starvation conditions, which could be explained by different levels of repression of Oct-1 in these two scenarios. Moreover, the presence of accumulated prelamin A in hMSCs under stress conditions could alter other transcription factors and/or pathways otherwise intact in Oct-1 silenced hMSCs. These results are supported by a previous publication [58] in which the authors described a pathological sequestration of the transcription factor SREBP1 by prelamin A at the nuclear envelope in fibroblasts from laminopathy patients and in murine pre-adipocytes. More recently, our group has previously described that accumulated prelamin A interacts physically with another transcription factor, Sp1 in adipocytes derived from hMSCs, thereby altering their functionality [23].

In summary, we have demonstrated that the accumulation of prelamin A in hMSCs induces a premature aging phenotype which ultimately results in a clear decrease in its functional capacity *in vivo.* In this human experimental model of premature aging, the aged phenotype is clearly exacerbated when the prelamin A accumulation is combined with a stress condition as has been observed by means of increased DNA damage, impaired autophagy, significant gene expression changes and Oct-1 impaired activity. These results are not identical to those observed in the progerin expression based models where the mere presence of progerin at levels found in patients is enough to provide the severe phenotypes observed in the absence of stress conditions. These facts are consistent with the different severity of the phenotypes observed in *LMNA*-linked lipodystrophy (early onset aging) and in progeria patients (late onset aging) indicating the value of our model to the study of early onset of aging. The more human experimental models created specific to varying degrees of aging (from early to late onset), the more complete the characterization of the physiological aging process will be.

Finally, the potential of this human experimental model has been demonstrated by the identification of a relevant role of Oct-1 in the aging of human mesenchymal stem cells, a function unknown until now.

## MATERIALS AND METHODS

### Cell culture

Human bone marrow derived mesenchymal stem cells (hMSCs) were obtained through altruistic donations from the Inbiobank node of the Spanish National Stem Cell Bank (www.inbiobank.org). Induction of prelamin A accumulation and cell culture was performed as described previously [23]. To induce serum starvation conditions, hMSCs were cultured in FBS-free medium during the last 24 hours of culture.

### Telomere length analysis

Telomere length was analyzed in hMSCs derived from 4 different bone marrow donors by using the HT-Q-FISH technique (Life Length). At least 17,000 nuclei per sample were analyzed ensuring reliable measurements of mean telomere length and the detection of nuclei with short telomeres (< 3 kb).

### Immunofluorescence

hMSCs were grown on glass coverslips and fixed in paraformaldehyde 4% (Sigma). Immunostaning was carried out using the following antibodies: anti-phopho H2AX (Millipore), Oct-1 and prelamin A and β-tubulin (Santa Cruz). DAPI (Sigma) was used to counterstain cell nuclei.

### Hypoxia

For hypoxia experiments hMSCs were exposed to 4 hr of hypoxia via mineral oil immersion followed by reoxygenation in normal conditions for 24 hr. Surviving cells were counted using Trypan blue stain (Gibco).

### ROS measurement

To detect the production of ROS 1.10^6^ cell/mL were resuspended in PBS 1X containing the fluorescent molecule CM-H_2_DCFDA (Molecular Probes) at a final concentration of 3 μM. Cells not stained were used to determine auto-fluorescence and deducted from all experimental data. hMSCs were acquired in an EPCS XL Flow Cytometer (Beckman-Coulter).

### Animals and surgery

Limb ischemia was induced in severe combined immunodeficient (SCID) mice (12 weeks) (Janvier). Animal studies were approved by the animal ethics committee of CIC biomaGUNE and conducted in accordance with the Directives of the European Union on animal ethics and welfare. Mice were anaesthetized with 4% isoflurane and maintained by 2% in 100 % O_2_ and left hind limb ischemia was induced. SCID mice were immediately assigned to the following experimental groups. A total of 3x10^6^ of cells in a volume of 200 μl or medium (DMEM) was injected intramuscularly into 4 sites of the gracilis muscle.

### Measurement of hind limb blood flow

Hind limb blood flow was assessed with a laser–Doppler system (Perimed AB). The laser-doppler small straight probe (∅ 1mm) was placed over the mice feet to measure the superficial blood flow for 10 minutes. For the quantification, the acquisition was averaged and expressed as the ipsi-to-contralateral hind limb ratio of arbitrary units.

### Radiochemistry

For the production of [^13^N]ammonia, a 5 mM aqueous ethanol solution (1.7 mL) was bombarded with 18 MeV protons (target current= 20 μA) in an IBA cyclone 18/9 cyclotron (^16^O(p, α)^13^N nuclear reaction). The irradiated liquid was eluted to selectively retain [^13^N]NH_4_^+^. After washing the cartridge, the desired radiotracer was eluted with 10 mL of physiologic saline solution.

### PET scans and data acquisition

PET scans were performed 14 days after the induction of hind limb ischemia in mice using a General Electric eXplore Vista CT camera. Anaesthesia was induced and animals were placed into the PET. Around 50 MBq of [^13^N]NH_4_ was injected in a tail vein 10 minutes before the start of the PET acquisition. Brain dynamic images were acquired for a total of 22 minutes, providing 175 transaxial (0.387mm thick) and 61 axial slices (0.775mm thick). After each PET scan, CT acquisitions were also performed. Dynamic acquisitions were reconstructed with filtered back projection (FBP) using a Ramp filter with a cutoff frequency of 1 Hz.

### PET Image Analysis

PET images were analyzed using PMOD image analysis software (PMOD). The volumes of interest (VOIs) were manually defined in each animal from the iliac crest until the end of the ischemic and contralateral hind limbs by using the CT images. For quantification, frames 27-30 last 10 min of PET acquisition were averaged and the uptake in each VOI (mean±standard deviation) was determined and expressed as the ipsi-to-contralateral hind limb ratio of percentage of injected dose per body weight (%ID/g).

### Microarray hybridization and data analysis

Microarray experiments and expression data were performed and analyzed as described previously [23]. Two biological replicates and one technical replicate were characterized per treatment: thus, three independent hybridizations per treatment were performed.

Microarray data were deposited at Gene Expression Omnibus (*GEO*, www.ncbi.nlm.nih.gov/geo) with accession number GSE52563.

### Functional annotation and transcription factor binding analyses

The dysregulated genes were analyzed using the annotation Database, Visualization, and Integrated Discovery (DAVID) gene ontology (GO) database (http://david.abcc.ncbifcrf.gov) taking into account a background that contained all genes present on the human HT12 v4 Illumina microarray. For this analysis, genes with a fold change > 1.4 or < −1.4 (p-value < 0.05) were considered to be regulated. Statistically over-represented GO terms were identified by selecting those with an EASE score (a modified Fisher Exact Probability P-value) < 0.05.

To test a possible enrichment for transcription factor binding sites within the promoters of dysregulated genes the DiRE server (http://dire.dcode.org) was used [39]. “Occurrence” indicates the percentage of candidate regulatory elements containing a conserved binding site for a particular transcription factor, and “importance” is the product of occurrence and weight, which in turn is a measure of the association of a transcription factor with the overall input gene set.

### RNA isolation, reverse transcription and qRT-PCR

Total RNA for each sample was isolated with High Pure RNA Isolation kit (Roche) and 1 μg of RNA was reverse transcribed using the High Capacity cDNA RT kit (Applied) according to the manufacturer's instructions. All reactions were carried out at minimum in triplicate on an Applied Biosystems 7900HT Fast Real-Time PCR System using Power SYBR Green PCR Master mix (Applied Biosystems). For gene expression normalization *GAPDH* was used. Primers sequences are available upon request.

### Western blot

Cellular extracts were loaded into 4-12% Nupage Novex Bis-Tris Mini Gels (Invitrogen). Primary antibodies used were the following: pS6 and total S6 (Cell Signaling), LC3 (Pierce), p62 (Pierce), Oct-1 (Santa Cruz), and β-actin (Sigma). SuperSignal West Pico or Fempto Chemiluminescent Substrate developing kits (Thermo Fisher) were utilized to develop signal. For quantification, ImageJ program was used.

### Luciferase reporter assay

400,000 hMSCs were nucleofected (Amaxa Biosystems) with 2 μg of pOct-1-Luc reporter vector (Signosis). Transfections were performed in triplicate and cells from each transfection were seeded in a well of a 6-well plate. 24 hours after nucleofection, cells were starved for 24 hours. Transfection efficiency was determined by co-transfecting with Renilla luciferase control vector (Promega) per transfection. Firefly counts were normalized against Renilla counts and triplicate samples were then averaged.

### Lentiviral vectors generation and hMSCs transduction

Lentiviral constructs NT shRNA pLKO.1 and Oct-1 shRNA pLKO.1 were obtained from Sigma. Lentiviral vectors were generated at the Viral between passages 7-8 were infected at multiplicity of infections (MOI) of 10 in the presence of 8 mg/ml of polybrene.

### Statistical analysis

Unless otherwise stated, data are derived from at least three independent biological replicates on triplicated samples and expressed as means ± standard deviation. The Mann-Whitney U test was used to determine statistically significant differences and p<0.05 was considered to be statistically significant. In the case of telomere length Student's T test was used.
